# Scarcity, Property Rights, Irresponsibility: How Intellectual Property Deals with Neglected Tropical Diseases

**DOI:** 10.1007/s10978-022-09324-3

**Published:** 2022-06-25

**Authors:** Daniel Pinheiro Astone

**Affiliations:** Faculty of Law, Stockholm University, Stockholm, Sweden

**Keywords:** Scarcity, Irresponsibility, Intellectual property, Neglected tropical diseases, Public domain

## Abstract

The article addresses the role of scarcity in negotiating the relationship between intellectual property, particularly from a legal-economic perspective, and property rights, as understood by transaction cost economics, to shed light on the deadlock faced by those suffering from neglected tropical diseases (NTDs). The consistency of the law and economics fundamentals that support the trade on knowledge goods, namely patents on essential medicines, is put under check by Scott Veitch’s scholarship on legal irresponsibility. The damages that emerge from the operations of the intellectual property system are registered in the novel concept of negative public domain, and are due mainly to the lack of access to treatments that end up being unaffordable, or to innovation that leads to new drugs that is not sufficiently incentivised though price signals. The accountability for such damages is taken into consideration by arguing that the disavowal of responsibility is made possible by the negative public domain, which is balanced by the construction of a positive response through the language of rights. As such, responsibility per se is preserved, evading one instantiation of Teubner’s legal paradoxes, but rendered ineffective by design. In other words, even if the harms endured by those affected by the NTDs can be traced back to the operations of the intellectual property system, there is no one to hold accountable. The main goal pursued through the article is to make such an arrangement explicit, by giving centrality to the notion of scarcity and its interplay between legal and economic theory, alongside the novel concept of negative public domain as a site where the actual consequences of irresponsibility lie, to hopefully inform further critique in subsequent works.

## Introduction

Scarcity is a powerful concept. It informs the core of economic science, rendering substance to the very possibility of allocation in the first place. It drives prices and makes the idea of efficiency meaningful. On the flip side, it denotes lack, poverty, and inequality. Using scarcity as the conceptual and linguistic bond between these two dimensions, this paper offers an initial attempt at studying how the booming intellectual property system and the long-standing issue of neglected tropical diseases can co-exist in a globalised economy.

To explore the plasticity of the scarcity concept in the international arena, this paper looks at the issue of the neglected tropical diseases (NTDs) for both its horizontality — those affected by these diseases are bound together due to circumstances of extreme poverty (scarcity as *lack*) — and its vertical unfolding: innovation in the pharmaceutical sector occurs mostly in the Northern hemisphere, and products are patented by private companies driven by profit (*induced* scarcity) before reaching those in need elsewhere. Moreover, the intellectual property system, operating under the Trade-Related Aspects of Intellectual Property Rights (TRIPS) Agreement, adopts legal tools to interfere with the nature of knowledge, an otherwise non-scarce resource, to induce its scarcity with the self-declared purpose of generating incentives for innovation and ultimately of creating value. In doing so, the system veers away from those affected by NTDs, precisely because these persons lack the means to trigger the incentives devised within the Intellectual Property (IP) system.

The enduring tragedy is evident and there are several responses in place, from philanthropy to patent pools to state intervention. Still, there is no attribution of responsibility. If it is accepted that the IP system, as it stands today, is a result of struggles of power(-knowledge), with winners and losers, then intuitively the damages would generally point back to their perpetrators — those at the other end of an institutional causal link. However, as Veitch argues, this is not the case (2007). For him, whenever law assigns grounds and boundaries for responsibility, it also defines the conditions for irresponsibility. The goal of this article, then, is to structure a discussion about how this articulation between material and induced scarcity is organised through law, while the resulting harms from its operations are left without proper instruments of accountability.

As such, the discussion here focuses on the normalisation of scarcity from a legal-economic perspective, concerning the subjects of access to medicines and NTDs. It starts with a thorough understanding of the concept of property rights and its intersection in law and economics to connect the widely accepted economic notions gathered from authors such as Yoram Barzel with Scott Veitch’s critical analysis of legally attributed (ir)responsibility. From the former, it is of particular interest to explore the creation of property rights towards elements of the public domain, especially non-rivalrous goods, as in the case of knowledge. The latter informs the legal approach used to study the consequences of such enclosures, in both practical (in terms of denial of access and the dynamics of market transactions) and theoretical terms (in conceptualising justice and registering the corresponding material consequences).

The work of classical political theorist John Locke posits that scarcity emerges from the attribution of privately owned property rights over a given set of goods, justified as a necessary condition for progress. For knowledge goods, scarcity derives from the attribution of intellectual property rights and their exclusionary effects, granted to enable owners to recoup investments and enjoy the inherent incentive of profit (Posner 2005). On patented drugs, however, this may result in situations where treatments are available but remain inaccessible if there is no compliance with the conditions established by the patent owner, particularly paying the asking price.

Halting investigation at this point would lead to a normative pitfall involving the various flavours of justice and resulting political choices (Drahos 2016). Conversely, scrutinising some of the canonical assumptions of IP could lead to an effort to project and propose contingent alternatives (Kapczynski 2016). While acknowledging these important contributions, this article uses the debate raised by Veitch to examine NTDs and access to medicines, focusing on the normalisation of scarcity through the lens of responsibility. This could eventually point towards a system for which no one can be held accountable. If this is so, the existing intellectual property system could imply a certain pre-justice arrangement — one that endures as long as the structure supporting the market economy, and in particular intellectual property rights, can maintain an element of neutrality (or normalisation). In this sense, if harm is in fact endured by people deprived of access to medicines or drug innovation, the public domain could be encumbered by externalities emerging from scarcity, resembling a zero-sum system, while also exempting actors from the consequences of individual actions.

Still, this framing of the subject does not forcefully translate into a problem. After all, the foundation of responsibility is legal. But in dealing with normalisation as a process, it is necessary to decouple the epistemological link formed between law and justice, implicitly operated through the canon of rule of law (Derrida 1992). It is not the goal of this article to advance what normative goal could or should be achieved through the patent system, but rather to analyse it from a sufficiently broad perspective based on a certain level of agreement between different theoretical affiliations, which is the necessity of at least *some* level of social justice as a condition of property in itself.

Consequently, the normative canvas is rather agnostic, meaning there should be some level of reciprocity if social actors are to accept intervention in individual freedom, in the form of arbitrary attribution of property rights over goods that would not be scarce in the state of nature. Pinpointing and defining such reciprocity is up to the reader. If one wants to stay closer to the classic liberal inspiration that informed the economic theory mentioned in this article, Merges (2011) offers a feasible account of reciprocity in terms of the Lockean proviso of charity. On another spectrum, challenging a certain impunity that characterises the attribution of property rights in the first place, it is possible to rely on Davies (2020) and seek the justification of property as an exception that can stand only if the collective benefits by doing so. Eventually, to stay closer to the critical analysis developed here, it might be beneficial to rely on Veitch’s (2021) own discussion of obligation as the counterpart of rights discourse — as something akin to duty — so that a property right would entail a form of property duty.

The timing of this study is noteworthy. The first draft was produced by the end of 2019, before widespread knowledge of COVID-19. Since then, academic and non-academic circles have had recurring discussions about patents on vaccines and essential medicines, funding of socially relevant scientific research, the geopolitics of intellectual property and the close ties between rich countries and domestic pharmaceutical companies, and most of all, the need for state-led action to provide a meaningful response to mass health concerns. In rough numbers, COVID pandemic statistics are not very different to those for NTDs. Still, a wide variety of solutions and enormous amounts of public funds were mobilised in the search for a cure. While this article does not analyse the pandemic directly, COVID-19 stresses one of its key arguments: NTDs are an ongoing calamity, basically because those affected by them are invisible *by design*. As discussed in the following section, it is in the foundations of the intellectual property system to ignore the needs of those who do not express themselves through price signals, which in this case are those who are *unable* (and not those who are *unwilling*) to pay for treatments: the extremely poor individual, the neglected group of people, the economically irrelevant state.

The article is structured as follows. Section 2 sketches the background offered by mainstream intellectual property theory, transnational imbalances that emerge from the global trade in knowledge goods, and their circularly cumulative relationship with poverty. Section 3 critically assesses scarcity as the link between transaction cost economics and intellectual property theory, further exploring the links between the two and advancing a framework for addressing responsibility for instantiations located outside the scope of market transactions. Section 4 focuses on the four pillars that inform the critique: (i) property rights as an analytical tool to inform legal analysis, (ii) a problematisation inspired by Veitch’s scholarship on law and irresponsibility, (iii) further discussion of the negative public domain — which acts as a register of the costs incurred by the purported value creation advanced by property rights theorists — and (iv) a final remark on intellectual property and commodification. The last section revisits previous arguments on the recognition of harms and the subsequent disavowal of responsibility without reaching the legal paradox of damage without acknowledgement. It is suggested the register of legitimate expectations into the language of *rights* works to preserve the stability of legal reason while obstructing the assertion of responsibility as a duty derived from obligation, which is rooted in a system of values. The main goal of the article is to make such an arrangement explicit, particularly by giving centrality to the notion of scarcity and its interplay with law and economics, alongside the novel concept of negative public domain as a site where the actual consequences of irresponsibility lie; it is hoped that this will inform further critique in subsequent works.

## Intellectual Property Theory: Canons and Economic Justifications

The benchmark for the subject in the international arena is the Agreement on Trade-Related Aspects of Intellectual Property Rights (TRIPS), presented as a document that ‘frames the IP system in terms of innovation, technology transfer and public welfare […] a legal recognition of the significance of links between IP and trade and the need for a balanced IP system’.^1^ The idea of a *balanced* system is as contradictory as it is informative in terms of the hybrid legal and economic critique advanced in this article.

If innovation is called into question under the canons of intellectual property theory, it is possible to argue, following Posner, that the intellectual property system works by providing incentives to innovation based on the possibility it grants to creators to temporarily restrict access to their creations (Posner 2005). This prerogative enables charging of prices that exceed the marginal costs, a circumstance equivalent to a time-limited monopoly. In other words, scarcity in this case is *induced*, in the sense that knowledge would otherwise circulate freely without accruing to the respective creators. By making knowledge scarce, Posner and Landes explain, incentives are created and subsequently preserved, avoiding the once-again invoked problem of the tragedy of the commons (2003, pp. 223–224). Following this track, it is possible to say that innovation can be conceived as a function of potential profit. It informs (neoclassical) self-interested agents, who would otherwise divert resources to activities that could better maximise utility. As the standard theory goes, society as a whole improves when knowledge is made scarce (Haber 2016), by framing knowledge as ordinary (albeit intangible) goods to incentivise innovation. The opposite – knowledge goods readily available to all, regardless of the development costs – not only fails to provide adequate incentive; it would actually discourage inventors.

Still within the field of conventional IP theory, Merges argues that ‘individual control over individual assets [is] the once and future essence of [intellectual] property’ (2011, p. 295). For him, distributive justice is already fulfilled through patent term expiration, exceptions, and tax incentives, and by owners voluntarily deciding on the nonenforcement of patents. As Lemley pointed out, Merges recognised there was insufficient evidence to support intellectual property law, and that Merges’ own theoretical production disregards the lack of empirical elements to support IP as a matter of faith (2015, pp. 1336–1337). He draws from Locke regarding the justification of intellectual property rights as something borne out of ‘[t]he relationship between labor, appropriation, and human flourishing’ (Merges 2011, p. 40), whenever labour is mixed with knowledge dispersed in the public domain (Merges 2011, pp. 42–44). For him, as for Locke, it is not any labour that enables property rights to be ascertained; it must be useful, thus contributing to human flourishing; it is precisely this element that is contained in the non-obviousness requirement of a patent award (Merges 2011, pp. 58–59). Merges also questions whether it is at all useful to try and answer what he calls IP’s Big Question — whether patents are justifiable on economic grounds — positing that this elusive effort should give way to more discrete and localised analyses (Merges 2017, p. 200).

This article argues that this elusiveness does not necessarily exist. As described below, evidence abounds in the sense that the economic grounds of intellectual property, whatever one defines as strictly *economic* for that matter, are as fragile as their definition is biased in the dominating narrative (Boyle 2008; Lemley 2015). The flip side of the merry story found in IP textbooks is not one of countries following the trend of so-called advanced economies, joining the global market under the TRIPS standards, and experiencing steady, privately led improvement of their internal indicators. Instead, the tale is a harsh one, where poor countries suffer from costly and inaccessible patents for critical technology (Müller-Langer 2009), substandard medicines pose a life threat to patients as a consequence of the permanent pursuit of lower production costs (Caudron et al. 2008), and generation after generation of people becoming trapped in a poverty cycle that not only affects their immediate economic outcomes but acts as a poverty-promoting force (Hotez 2013, pp. 11–12).

## A Critical Assessment of the Idea of Scarcity

As briefly noted earlier, the NTDs encompass a set of twenty diseases that affect one billion to two billion people worldwide (Zhang et al. 2010), meaning there is an obvious demand for treatments. It is estimated almost every single person in the so-called bottom billion has or has had an NTD, and that these persons live throughout 56 of the 58 countries where people in the bottom billion live (Hotez et al. 2007, p. 1570). Still, this demand for treatment remains unfulfilled, be it in terms of innovation, access to existing medicines, or other policies related to basic living conditions (Choffnes and Relman 2011). Their socioeconomic consequences are pervasive. Observers tend to highlight the economic impact in terms of opportunity losses to suggest cost-benefit prospects in offering mass treatment at a relatively low cost (Holmes et al. 2017), which might then serve the purpose of seeking immediate care through strategic partnerships and diplomatic roundtables, for example, to establish donation agreements with global pharmaceutical companies. Even more important, though, are the causal, mutually reinforcing, and long-term links between NTDs and poverty, the disproportionate burden that NTDs pose on women and children, and the illnesses’ developmental and cognitive effects (Hotez et al. 2009).

It might be trivial (but nevertheless disturbing) to suggest that people living in areas where most basic resources are scarce are unlikely to offer sufficient market incentives to trigger the economic interest of the pharmaceutical sector. In fact, in sub-Saharan Africa, which is the most affected region in the world in terms of NTDs, 51% of the population lives on less than USD 1.25 per day and 73% on less than USD 2.00 (Mitra and Mawson 2017). Conversely, there is no legally informed rebuttal to the fact that this condition will likely persist as long as no market incentives are present to trigger an innovative response from companies; in other words, suffering in itself has no legally recognisable causal effect in relation to the underlying structure designed to provide means for disease alleviation (Veitch 2007).

As discussed above, it is economically rational and legally acceptable for companies to divert their scarce resources according to their own strategic objectives, e.g., towards more utility-maximising activities. For instance, some authors argue for stronger patent protection for industry leaders to favour a trickledown effect in terms of innovation (Acemoglu and Akcigit 2006) or full discretion in establishing prices and profit targets for relevant medicines to maintain the relative attractiveness of R&D efforts (Sonderholm 2009). Others assert that the net costs of any increase in patent protection terms (at least regarding medical treatments) would ultimately be less efficient than using the same resources within the scope of proper healthcare policies (Outterson et al. 2007).

Among the twenty diseases in the 2020 version of the list maintained by the World Health Organization,^2^ a few are treated using well-known drugs that are still not universally available (e.g., malaria) and others that required significant time to attract substantial investment to fund research and development (e.g., dengue). As these diseases hardly characterise a standard market able to attract the interest of the main pharmaceutical companies, it is no surprise that the investment in treatments is intermediated by transnational structures (e.g., the WHO itself). It is worth noting that these structures are generally publicly funded — either directly, as in the case of governmental transfers coming from taxation, or indirectly, as in the case of philanthropy and other forms of tax-exemption mechanisms (Kapczynski 2016). It has been recurrent to discuss the problem in terms of costs and benefits, or even the loss of productivity experienced by those exposed to NTDs (WHO 2002).^3^ Once again, suffering in itself does not necessarily imply rejection; in fact, suffering is accepted as a consequence of the attempt to strike a balance and find a manageable level of suffering vis-à-vis its costs, while still enhancing or maximising the economic outcomes of a given social group (Mbembe 2019).

Since NTDs are causally related to poverty (Choffnes and Relman 2011), the elasticity of corresponding treatments is likely limited by material circumstances in a way that makes it impossible to match demand and purchasing power. This means that even if demand is markedly high, its translation into prices will be insufficient, as it would be vocalised through the price signals emitted by the extremely poor, who naturally lack capital to send sufficiently attractive signals (Stiglitz 2008). This notion could be conceptualised as *price-as-voice*, and could even include public procurement if one accepts that a state’s income is at least partially informed by the net wealth of its population. As such, insofar as innovation under the current intellectual property standards is responsive to market signals, and as NTDs consistently reduce the economic perspectives (not to say overall life perspective) of poor peoples, a self-reinforcing effect would hamper further efforts to becoming autonomously included in an IP-governed system.

It seems there is an immanent contradiction (Christodoulidis 2019, p. 7) between the exceedingly high demand for medical treatments for NTDs and the lack of incentives able to trigger innovation. It is proposed that at the centre of this contradiction lies the bi-dimensional notion of scarcity (both as *lack of resources* and *induced scarcity*). Price (signals) vocalises relative demands in a context of scarce resources. On the one hand, companies will answer to such signals to mobilise resources that are naturally limited and will be lacking somewhere else once consumed, such as workforce, intellectual capacity and infrastructure. On the other, the measurable output will be artificially scarce, as it will result in a potential finding or invention that could be subject to patenting. These will then be offered in a market which speaks the language of prices as a medium of exchange, as a reductionism to all other naturally scarce resources that are nominated under the label of a given currency. While this certainly lower transaction costs and uniformises communications, it is quite obvious that the ability to ‘emit’ price signals is far from equally distributed, as it derives from relative wealth — hence the analogy of *price-as-voice* to stress the idea that, by design, the intellectual property system cannot adequately listen to and register the demands of people distributed worldwide in equal terms.

Normative implications emerge whenever one throws light on the assumption of price signals acting as a proxy for actual needs. Even if it feels inherently wrong to argue the possibility of seeing people die because they lack access to essential medicines, arguments to the contrary provide reasonable grounds as support (Sonderholm 2009). *Someone* must pay the price of drug development, and under the current framework, the scope for alternatives is very limited. Development costs are currently recouped through sales or licencing prices — aspects closely related to *price-as-voice* — throughout the 20-year patent term. From this perspective, the only concrete means for drugs to be developed in the first place is to grant the time-limited monopoly, thus making generic drugs possible in the future. This raises three additional problems. From an ethical standpoint, it normalises the possibility of letting people die today, say for lack of access to medicines, to save people tomorrow. However, the selection of those who are expected to die is far from horizontal: those who will die are the ones who cannot afford drugs at monopolistic prices. As such, patents are saving *some* today at the expense of others. In some ways it is a tax on life – an extremely regressive one that takes poor lives to save rich ones, while promising those on the losing end that the future will be better. The second problem is that this future might never happen. As shown by Feldman in a survey of all the drugs available in the market from 2005 to 2015, companies actively employ strategies to extend patent protection well past the 20-year term. In fact, Feldman identified that 78% of the new patents covered existing drugs, a practice referred to as evergreening (Feldman 2018). Finally, this approach does not solve the innovation problem. If monopolistic prices stand as the main incentive to drive drug development, new drugs must be commercially viable today to enter the public domain tomorrow. In other words, there must be a market now — one willing to pay high prices — and this market is certainly not populated by the NTD-affected population.

As such, normalising the assumption that patent protection is an imperative for drug development, as Sonderholm (2009) argues, implies exempting those involved and the patent system in itself from any responsibility for the harms caused by denial of access to medicines. The discussion of whether such a system is *just* tends to lie outside conventional legal and economic scholarship; after all, it is perceived as a *political* matter (Okediji 2018; Whyte 2019). Challenging such normalisation can possibly achieve more than just re-stating the obvious — that people are dying from the lack of essential medicines and companies are not to blame — if there is an effort to dig deeper into the notion of public domain as it is portrayed by conventional IP scholars.

As discussed in greater length in the following section, the public domain is ordinarily portrayed as a dispersed repository of valuable resources that are waiting for propertisation to become useful. The prevailing discourse has been that well-defined property rights allow entrepreneurs to protect their efforts in innovating and improving such resources, thus creating value at some point in the future. Conversely, the unorganised usage of resources found in the public domain would lead to depletion, so that the only way of benefiting society with innovation is by protecting incremental efforts of innovation with intellectual property protection (Merges 2011). However, such authors refrain from addressing what actually happens once the public domain is subject to commodification. Restriction of access may quite naturally protect investments, but what is the cost faced by those on the other side of the fence, such as those that may want access to an essential medicine or treatment, for instance, but do not have means for paying discretionary costs established by the owners of patent rights?

To provide an analytical container for such costs, this article argues for a wider notion of public domain — one that could act as the bearer of the costs derived from scarcity, for instance as implied from the attribution of intellectual property rights, or in other words, the damages resulting from the many aspects implied in the concept of scarcity. If there is indeed no implied responsibility once the mechanics of property rights are normalised, there is no one to blame. It seems especially informative, then, to explore the interplay of the attribution of property rights over the public domain, to the consequences for the public domain once parts of it are subject to capture, and the role of legally attributed *irresponsibility* in organising and normalising such a structure.

## Approaching Scarcity and Irresponsibility

This section offers an analysis of the key inputs from transaction cost economics that inform intellectual property theory, with a focus on two aspects. The first aspect is the understanding of a property right as an equivalent to the prerogative of inventors (or of the rights owners who accrued the inventor’s residual rights), which unfolds first as *ability* then as *legal claims* over something. The second aspect changes the vantage point to examine what happens to those who are subject, directly or indirectly, to the exercise of these same property rights. In other words, as proposed by Coase (1960), the exercise of any right occurs at the expense of a third party, so it seems natural to examine the circumstances experienced by those who no longer have rights over goods that they would otherwise have the *ability* to use.

### Transaction Cost Economics and Property Rights

It is generally accepted that law allocates responsibilities, encompassing decision rights, entitlement, liability, conventional property rights over goods and commodities, and so on (Barzel 1997; Coase 1960; Eggertsson 2009; Williamson 2010). These rights fulfil an organisational demand characteristic of modern societies by allocating these responsibilities in a rational, positive, aspiringly scientific manner (Barzel 2001, pp. 176–178). Arguably, then, there is a positive functional aspect pursued through law in creating, sustaining, and enforcing hierarchies based on the allocation of rights, which entails a specific and restricted range of responsibilities.

For Yoram Barzel, the state is fundamentally a Hobbesian construct that derives from the cost-benefit analysis of individuals seeking to maximise their utility beyond the limits imposed by the preceding state of nature (Barzel 2001, p. 2). He argues that, in the absence of a protector, assets are potentially subject to theft, which amounts to ‘a transfer of wealth at resource cost’ (Barzel 2001, p. 158). The notion of assets includes not only physical assets and intellectual creations but also self-ownership (e.g., the individual herself but also others, such as slaves) and reputation (Barzel 2001, p. 157). As it stands, benefitting from one’s labour without adequate compensation would result in theft as much as stealing an apple.

This holds even for knowledge goods, where their capital value rests precisely in their induced scarcity, which will cease to exist once the goods are ‘stolen’. The thief, on the other hand, will not bear the costs of the exclusivity imposed by patent law, and enjoys such assets at the bare minimum cost of acquisition and usage, the so-called resource cost. Conversely, the state acts not only as the protector but also as the entity that legitimately determines those who can exert institutional violence to protect property rights, while it is also encumbered with the competence for assigning property rights to minimise the costs faced by social actors in protecting wealth and promoting adequate transactions.

Quite obviously now, the key conceptual tool found in transaction cost economics is *property right*. This right has two distinct yet intertwined meanings in economic literature. The traditional one relates to rights that the state assigns to a person, i.e., when determining that one is the owner of something. The other relates to the *ability* one holds to exercise rights over the corresponding property (Barzel 1997, p. 3). In other words, Barzel posits that ability is the main feature defining *economic* rights — meaning the ability to enjoy one’s rights, regardless of their legality (Barzel 2001, p. 6) — which he then distinguishes from *legal* rights, which are those derived from state power. The latter can also be defined as ‘[…] the claims over assets delineated by the state as the property of particular individuals or institutions’ (Barzel 2001, p. 157). As such, legal rights are not a condition for property rights *per se* but may supplement them by creating artificial boundaries to undesirable (arguably inefficient) economic rights.

A question can emerge when one tries to distinguish economic and legal rights whenever the underlying object is an intangible good, such as a patent. It is only possible to exert any right over a patent once the patent itself is created as an effect of law. This could lead to the idea that there are no rights except legal rights, as such rights are a fundamental prerequisite for ability, the chief feature found in legal rights’ economic counterpart. Albeit compelling, this rationale is consistent with the theory only at a micro level, in dealing with the patent as a given. For example, an agent might have the technical capacity to reverse engineer something; this person would have ability, or the economic rights to do so, as a representation of the resources at his or her disposal to do so. However, intellectual property provisions render the act unlawful. The role of legal rights as state-informed boundaries becomes increasingly clear as one moves up the scale towards further abstraction, for instance in examining not a patent but a discrete portion of innovative knowledge. Unencumbered by patents, these pieces of knowledge could be acquired by everyone once they are made public. In strict terms, everyone has an ability, as related to Barzel’s economic rights, to access and use such goods. Incremental usage would not make these goods wither away (a characteristic of a non-rivalrous good) while they would also be universally available, with no limit to their supply (a non-excludable good). In other words, knowledge would still be part of the public domain, not as something ancillary to the patent system but as a domain of all and for all (Boyle 2008). It is only through legal rights that pieces of knowledge are transformed into individualised assets.

Following this line, it is possible to argue that for Barzel, economic rights generally prevail over legal rights, although both comprise what he (and others) group under the concept of property rights, as the former exists irrespective of legal rights. Legal rights, however, can change the allocation of economic rights. Hence, when an intellectual creation (e.g., a patent) or the accumulation of wealth receives legal protection, the state in a sense is ruling out some economic rights to re-design incentives in line with a given public goal. In the case of essential medicine patenting, for instance, knowledge that could be readily available is made scarce through law. In such circumstances, legal rights reallocated economic rights over knowledge held in common to grant a monopolist ability over it; conversely, one could argue that the value of such an ability is related to the sum of the decreases on individual ‘wealth’ once potentially life-saving patents are made scarce.

There is an obvious caveat, which is the unlikely possibility that such patents would exist at all if property rights were not in place. This might be true under standards, although debatable due to the pervasiveness of public funding in basic research (Cross et al. 2021). The main goal, however, is to stress there is no value creation when property rights are designed; it is rather a matter of reallocation of rights and consequently, resources. What is particularly relevant as a takeaway from economic theory is that, first, the dimension of property rights refers to singular entities —the individual who has the ability to enjoy certain rights, the asset connected to such rights, the rights themselves (which can be alienated), and the (negative) ability or prerogative of rights owners to evade theft and exert (directly or indirectly) some type of violence against anyone who may potentially violate those rights. Second, at least for Barzel and other economists within the field, there is no normative implication associated with property rights analysis per se.

Three points are worth taking from Barzel’s scholarship. The first is that theft comprises a transfer of wealth without adequate compensation to the original owner. Second, violence/prohibitive action relates to an ‘impersonal means of imposing cost’ (Barzel 2001, p. 35). If the point of departure for him was a slightly revised Hobbesian state — one that not only monopolises legitimate violence but also has the power to assign third parties the authority to do so as well (Barzel 2001, p. 2). Specifically, this argument considers the ability of individual rights-owners to impose impersonal costs on others, with the aim of protecting rights-owner wealth. The emphasis here is on who should bear such costs — the costs derived from individually motivated violence — in the case of essential medicines. The third notable feature is the discussion of the role of property rights, particularly of the conceptualisation of legal rights, in dealing with responsibility. Barzel, alongside contemporaries (North 1990; Ostrom 1990, 2005; Williamson 2010), admits there is reasonable scope of potential states of equilibrium among different political choices made by a given social group. In other words, despite the generally sympathetic, liberal tone of these authors, they admit that a given rights arrangement is not given, but at least partially a result of choice. The discretionary nature of such enforcement carves the path for the discussion on Scott Veitch’s concept of irresponsibility, covered in the following subsection.

### Irresponsibility

As seen in the previous subsection, Barzel and transaction cost economists in general focused on a rather positive aspect of legal rights, stating that they are voluntarily asserted through state action to define and protect wealth. In a sense, they aim at expanding the scope of economic rights to make them positively identifiable.

From a different perspective, Veitch (2007) claims it is precisely by defining such legal boundaries or, in Veitch’s terms, in asserting *responsibility* that the legal system establishes grounds for *irresponsibility*. Hence, by defining what ought to be visible, law renders other elements of social reality invisible. In what could be referred to as a negative functional aspect — although not strictly through omission — he also claims that irresponsibility itself is organised, mirroring other instantiations of the social (Veitch 2007, p. 75). He argues that in a legal dimension, this has roots in a rule-making tradition reliant on generalisation, based on deliberately excluding realms, facts, and circumstances from a given set of legal scenarios or registers, enabling the expansion of the reach of the law, so the attribution of competencies and the demarcation of subject areas highlight aspects of the social that are deemed to be of immediate interest within a specific ideological framework. Accordingly, shedding light on the unseen parts of the intellectual property system brings the immanent problems of IP’s positive statements to the fore.

Looking at Veitch’s argument from another angle, the boundaries imposed by dogma are made explicit. Thus, ‘[w]here dogma does the work of sustaining the discursive space by setting in place the non-negotiable mainstays of the common, the work of legal hermeneutics is to unfold the dogmatic resources as interdiction and as judgement’ (Christodoulidis 2018, p. 5). Even when applying the critical framework as developed by Christodoulidis and Veitch, there is still a need for making irresponsibility operational at the same level of Barzel’s ideas as detailed earlier. If the law and economics discourse is generally susceptible to some level of legal reductionism (Christodoulidis 2018, p. 7), and Barzel is no exception, modelling irresponsibility in relation to property rights might emphasise what such a discourse lacks, or where its discursive boundaries are set.

For Veitch, ‘responsibility is a “normative device” and has been broadly conceived as, in some way, connecting answerability with agency’ (2007, p. 41). This situates this notion closer to Barzel’s *ability* while adding an element of causality. Being something *conceived* rather than naturally occurring implies a certain level of volition, of choosing among different arrangements so that there is no single structure for asserting responsibility, but rather competing (or at least plausible) *technologies of responsibility* (Veitch 2007, p. 39). In this interchange between the two authors, the terms of such causality answer to the legal infrastructure developed through state action whenever property rights are defined, attributed, and enforced; otherwise, there would be no answerability to emerge from agency.

Hence, for Veitch, once the legal dimension (of property rights) is delimited, say, within the framework that sustains property regimes (2007, p. 42), a second-order competency is attributed to organise the roles of its components. This suggests a connection with Teubner’s autopoietic dynamic of ‘law produced by law’ (Teubner 1993, p. 2). This stage is characteristically contractual, meaning it is unlikely that a broad share of responsibility will be allocated to a relatively small set of components; it is the sum of the individual(ised) attributions that will ultimately imply an outcome. There is scope for role responsibility, e.g., one that is limited to the particulars of a discrete position within a system, weakening recuperation of systemic or institutional responsibility. Therefore, it is generally not possible to track down discrete responsibilities based on a given systemic outcome. Each component deals with a small set of pre-established tasks and enjoys certain prerogatives that delimit the measure of lawfulness and, consequently, of (individual) responsibility (Veitch 2007, pp. 43–47). Hence, asserting systematic responsibility might demand identifying and re-establishing internal links from the bottom up, from the individual to the group, and finally to the leadership, and determining the adequacy of each instance of — returning to Barzel’s notion — ability, or at least bounded, legally-informed ability.

The dilution of systematic responsibility into compartmentalised tasks and attributions, each one obeying a set of formal criteria for validity, makes the case for the disavowal of responsibilisation, notably in instances where the damages might be evident but there is no instrumental route to reconstruction of causal links that would allow for reparation as we commonly conceive it. It is informative to consider that, as case studies highlight (Veitch 2007, pp. 8–12), the separation between systemic and discrete responsibility holds, even when the latter can be established.

Tentatively — and Veitch’s analysis is complemented here by inputs gathered from Teubner (1993) — it seems that the operations that shape the legal dimension of a given social activity, setting its boundaries, could be referred to as specialisation, established in rather than by law, in the sense that the legal form acts as a register for a certain area of expertise rather than as an autonomous source of competency. In segregating this space and providing a criterion for differentiation, specialisation delimits law in terms of reach. But delimiting may enhance depth imply the exclusion of different types of knowledge. The legal dimension does not carry the burden of being a perfect substitute for social life; rather, it openly seeks to systematise and articulate only those events that are deemed relevant to law and its purposes. If one accepts the scientific ambitions of Western law — of formal logic, practical reason, and generalisation — it may be deemed reasonable to account for some events but not others, as the opposite could result in an overly complex and impractical system (Teubner 1993). In other words, legal specialisation is selective, resulting in legal spaces that rely on the arbitrary acknowledgement of events, consequently, the conscious disregard of others.

### An Alternative View of the Public Domain

If the legal space does enjoy this relative factual autonomy, and if it is somewhat disconnected from the entireness experienced in the social reality, then issues such as reification and self-referentiality gain pre-eminence (Teubner 1993). The legal space could then be characterised as a function of specialisation *in* law, and as such, as realising itself *by* law, through following strict criteria of legal reasoning. Upon legally-based variables — instead of the corresponding factual ones — a set of meta-legal rules would organise relations between agents and events, or between events themselves. Regardless of the structure, the issue at stake is to observe how the specialisation level is organised by meta-rules, such as those found in jurisprudence, at a higher level of abstraction within the legal discourse, and as an effort of organising the legal space. If specialisation indeed entails abstraction to some extent, this might end up rendering some parts of the social reality excluded from the legal realm of observable phenomena. If this is the case, Veitch’s claims hold, as legal acts could be dissociated from material acts as long as the framework that informs legal causality is kept sufficiently separate from its underlying material consequences.

Hence, from Veitch we gather that from the set of agents and events, and the relations between these, it is possible to define two subsets: those that are legally recognised, and those ignored by law. Interestingly, the second subset resembles what Barzel referred to as the public domain. In saying that a ‘commodity lies in the “public domain” when the resources needed to acquire it accrue to no one’ (Barzel 1997, p. 5), it seems possible to transpose the core idea to analyse the responsibility for harms that are not legally accounted for. Even if there is no controversy over the existence of the damage in itself, it is not possible to attribute or causally relate it to a third party. Responsibility is dispersed, and tends to disappear, to the same extent as the resources spent in the public domain, even if someone endures the harm or acquires a commodity.

If material consequences do exist, nothing has in fact disappeared. Resources spent somewhere will leave a trace and at the very least will mean fewer available resources elsewhere. Harms endured by someone, even if imputable to someone else, will still be endured, have effects, and impose costs. Responsibility, as resources, could indeed be dispersed but would hardly disappear. What is called dispersion, then, could translate into a monitoring function explaining the apparent impossibility of restoring discrete relations of causality and instances of responsibility in each case. Irresponsibility, or what is left for the public domain in that regard, could be a consequence of high transaction costs that curtail the effort, as Veitch called it, of ‘connecting answerability with agency’ (Veitch 2007, p. 41). It could then become reversible to the extent that improvements would allow for better information, enforcement, price functions, and so on.

Eggertsson does not provide such a function but offers a plausible explanation in pointing to three factors capable of pushing ownership structures to what he calls common property and open access, which are conceptually equivalent^4^ to Barzel’s public domain. For both authors, these structures world as a repository for resources that are note privately controlled due to (1) high exclusion costs; (2) high internal governance costs when exclusive rights are shared; and (3) an open-access constraint enforced by the state (Eggertsson 2009, p. 264). Considering these factors, it is possible to draw a parallel to Veitch’s main arguments. Irresponsibility would occur whenever there are (1) high costs incurred in eliminating or avoiding harms; (2) high costs incurred to delimit, attribute, monitor, and enforce liability; and (3) legal grounds. Thus, irresponsibility would encumber a negative version of the public domain — a domain that accumulates the endured harms and welfare decreases that are left unaccounted for, instead of the free resources open to private appropriation found in this domain’s positive counterpart. It is a domain of disappropriation without accountability.

### Intellectual Property and Commodification

It is trivial to fit intellectual property rights into standard neoclassical reasoning, i.e. by assuming knowledge as something dispersed in the (positive) public domain, as a good that is gathered, processed, and improved by an agent, then commodified as a means to generate profit (Merges 2011, pp. 48–55). Continuing in this vein, we can say that if intellectual property rights were not in place, there would be no incentive to divert resources from other, more profitable activities, as the three previously identified factors would apply and every knowledge good would potentially fall into the public domain. Specifically, limiting access to innovation and medicines is necessary for the IP system to work; as discussed about, it is fundamentally reliant on limitations. As exclusion is inferred from its internal logic, and legally formatted and legitimised from a normative perspective — as it is normalised — there is no specific injustice derived from denying access to such knowledge goods; rather, it is a matter of faith (Lemley 2015). It is as if the original violence contained in exclusion were impervious to any consideration of fairness as the level of justification precedes that of law (Derrida 1992). One could anticipate that the removal of exclusion would not bring about reform but the collapse of the intellectual property edifice. This is neither just nor unjust; it is its condition of possibility. It is not anyone’s fault, yet it is everyone’s fault.

As advanced by this article, it is here that Veitch meets the neoclassical economists. In the attempt to account for responsibility, the analysis of the public domain unfolds in two. In commodifying knowledge, (1) the exclusion costs are immediately lowered, as they are now measured in terms of price; this occurs at the expense of others whose access is denied, so the costs of eliminating consequent harms could relate to forfeiting prices themselves. As for (2) governance costs, their reduction relies on contracts and courts as quintessential representations of the rule of law, obeying the limits of the respective legal space as a means to direct and protect resources that would otherwise dilute and risk expropriation; as the private regime takes the form of a legal space, specialised in scope and generic in relation to the social reality, what is outside this space ends up as a non-organised mass of public, anonymised liability, alongside resources and costs not yet commodified. Finally, (3) as the creation of property rights either outlaws or marginalises open access, the public domain becomes static or at least loses its fertility, in the sense that no incremental contributions would be made to the common pool of knowledge (its positive version), and the public domain is also encumbered by the social costs derived from the inherent exclusionary effects of intellectual property rights (its negative version).

Hence, low exclusion costs could imply high social costs as this necessarily translate into some form of alienation; low governance costs could imply the loss of nuance due to further narrowing of the legal space; and the creation of property rights could render the public domain less fertile, less valuable, and especially encumbered with the social costs derived from the previous cost reductions, because these costs would have occurred through denial of access. While neoclassical economists claim that the creation of property rights fosters productivity, innovation, and progress consubstantiated in prices and facilitated by lower transaction costs, Veitch calls into question the consequences of legally informed enclosure.

This last aspect is crucial if one observes the distinction between value and price. As already gathered from Barzel’s work, scarcity would emerge from an agent’s ability to enjoy the benefits of an exclusionary property right, but the measure of scarcity in terms of price — an absolute quantification based on currency — segregates its importance in terms of value; it is a relative quantification based on inherent criteria, such as labour or welfare. In other words, depending on the context, a small increment of work hours (e.g., at an average job in a rich country) would result in a higher increment of income when compared with the same number of hours expended by a poor worker somewhere in the peripheries of capitalism. Consequently, an under-priced commodity (e.g., a hypothetical patent) could potentially add value to the public domain, as the costs of development would be lower than its price without affecting its relative utility; however, considering value itself in relative terms, what is under-priced in the context of rich countries could well be radically overpriced in the context of their poorest counterparts. As such, it seems reasonable to argue that scarcity is regressively translational, meaning that the amount of value tends to increase as the relative wealth or income decreases. Poor people must *lack more* to have access to the same goods, including medicines (Stiglitz 2008). Thus, patents harm, but they can also save – for a price.

### Why am I being hurt?

In a book chapter dealing with dogma and obligation, Emilios Christodoulidis examines a gap: one that ‘opens between what is owed as obligation and what can be delivered as right’ (2018, p. 8). He retrieves an excerpt from Simone Weil, who proposes and discusses an eminently humane cry: *Why am I being hurt?* As she puts it, *apud* Christodoulidis, there can be mistakes in defining the harm, why and by whom it is inflicted, ‘[b]ut the cry itself is infallible’ (2018, p. 8). There is no question about the existence of a harm once it is endured, and once it is spelt out by those who are suffering.

Christodoulidis and Veitch converge in identifying a mismatch between the way that harms are registered, and the ethical and moral the grounds for asserting obligation/responsibility (Veitch 2021). They also agree on identifying the inscription of a mandate derived from obligation into the prospective category of rights. However, the subsumption of rights by a language of mediation erases the emergent possibilities of community and solidarity, and this creates a contradiction — yet again, an immanent contradiction — ‘between dignity promised and indignity delivered; between the promise of responsibility and the denial of fragility; between the promise of solidarity and the delivery of “shrill” contention and claim of right’ (Christodoulidis 2018, p. 11). Similarly, as detailed in the previous section, Veitch points out that the very design of such mediating language enables and promotes the possibility to disavow responsibility.

The interdiction posed by the language of rights as a means of dealing with human values and human needs is not new. Throughout the twentieth century, there was less of a dispute in terms of recognising arguably universal values than on agreeing on what to do about them (Anghie 2005), ultimately making a certain (rather market-friendly) version of human rights the main site for inscribing the demands of the poor, race, religious and gender issues, and so on (Moyn 2018; Whyte 2019). Put differently, it is not a matter of rejection of such demands but of contingent recognition of their terms. Doing otherwise could confront legal scholarship with a paradox (Teubner 1993, p. 11), creating a situation where there is suffering but no accountability. Rights serve as a register for such accountability — the demand for health and essential medicines is indeed recognised — but the operations that derive from such a prospective and general language are rendered useless; the actual demand is subsumed by another fundamental right, which is the right to property.

Access to essential medicines that disregards the rights of the patent holder implies liability and violates the fundamental right to property; conversely, *not allowing* access to essential medicines does not imply liability – it is simply a matter of exercising one’s rights. The first harm is measurable, identifiable, and punishable; the second harm falls into nothingness. As Veitch explained, whenever law organises duties and norms of conduct that characterise responsibility, it also organises — not through omission, but consciously — conditions for irresponsibility. And as was argued in this article, if this *nothingness* is indeed a place where all sorts of costs (of harms) can be imposed without consequence to the perpetrators, irresponsibility happens at the expense of the (negative) public domain.

There is a beautiful edifice built upon such language, and probably no more vivid depiction of this structure can be found than the one offered by Ursula K. Le Guin in her short story ‘The ones who walk away from Omelas’. Her allegory describes Omelas, a marvellous society where everything is balanced, merry and fruitful. However, this perfect world relies upon the extreme suffering of one neglected child. It is locked in the basement of one of Omelas’s perfect buildings – and Le Guin in fact refers to the child as an *it*. Every inhabitant of Omelas will, at least once in his or her life, encounter or become aware of the existence of this child. It is ugly, smelly, different, not as perfect as the Omelans. It is not clear whether the child is an Omelan or a foreigner entrapped in Omelas’s rules. However, the very fact that the happiness of the entire society depends on the suffering of this child renders most of its inhabitants consciously indifferent to the child. Alleviating its suffering would mean the beautiful edifice would collapse, and the happiness of all — or all but the child — would come to an end. For a few of the Omelans, the vision of the child eventually becomes unbearable, and they decide to act; as the title of the work suggests, they walk away. They leave child and society behind, untouched (Le Guin 1976, pp. 251–259).

Even in fiction, irresponsibility seems inescapable. But as Teubner suggests, it need not be that way, as paradoxes can and should be overcome in the work of the critic (Teubner 1993). Therefore, it is deeply meaningful to examine *neglected* tropical diseases in relation to intellectual property. They are neglected is a direct consequence of the asymmetry between the lack of those who endure suffering and the skewed allocation of resources to remedy it. It emerges from scarcity as the mediating concept in economic reason, a concept that translates value into prices and reacts to price-as-voice whenever one expresses demands through economic terms that were internalised by certain fields of law. Scarcity carries an inherent dimension of violence, while overall it is considered legitimate and regarded as a driver of progress. Because scarcity is normatively and legally justified, attempts to determine responsibility tend towards rejection. Still, the causal relations do not cease to exist, even if a given legal space ignores them and harms are most certainly endured, or if they are simultaneously a cause and a consequence of scarcity.

Mbembe (2019) demonstrated, this results from a system that accepts death and suffering as a natural consequence of its foundations. But at the current stage, any attempt at articulation of reform, policy prescription or any vague, rights-based motivational statement fall outside the scope of this article. Its only ambition lies in making the role of scarcity explicit, as an arbitrary legal-economic construct inflicting harms against human beings and against a revisited concept of public domain, by bridging the economic reasoning on property rights with the legal aspects of responsibility. The duty of the critic, then, is either to deal with a system that inflicts harms, and attempt to overcome its limitations, or walk away.

## Footnotes

1 Retrieved from https://www.wto.org/english/tratop_e/trips_e/trips_e.htm on 07 June 2021.

2 Retrieved from https://www.who.int/neglected_diseases/diseases/en/ on 07 June 2021.

3 This perception is not new. Foucault highlighted a similar framing within the public health discourse: ‘At the end of the eighteenth century, it was not epidemics that were the issue, but something else (…) illnesses that were difficult to eradicate and that were not regarded as epidemics that caused more frequent deaths, but as permanent factors which — and that is how they were dealt with — sapped the population’s strength, shortened the working week, wasted energy, and cost money, both because they led to a fall in production and because treating them was expensive’ (2008, pp. 243–244).

4 The equivalency was originally developed in Monteiro and Zylbersztajn 2012.
